# Crystal Structures of the Novel Cytosolic 5′-Nucleotidase IIIB Explain Its Preference for m^7^GMP

**DOI:** 10.1371/journal.pone.0090915

**Published:** 2014-03-06

**Authors:** Thomas Monecke, Juliane Buschmann, Piotr Neumann, Elmar Wahle, Ralf Ficner

**Affiliations:** 1 Abteilung für Molekulare Strukturbiologie, Institut für Mikrobiologie und Genetik, Göttinger Zentrum für Molekulare Biowissenschaften, Georg-August-Universität Göttingen, Göttingen, Germany; 2 Institut für Biochemie und Biotechnologie, Martin-Luther-Universität Halle-Wittenberg, Halle (Saale), Germany; University of Oulu, Finland

## Abstract

5′-nucleotidases catalyze the hydrolytic dephosphorylation of nucleoside monophosphates. As catabolic enzymes they contribute significantly to the regulation of cellular nucleotide levels; misregulation of nucleotide metabolism and nucleotidase deficiencies are associated with a number of diseases. The seven human 5′-nucleotidases differ with respect to substrate specificity and cellular localization. Recently, the novel cytosolic 5′-nucleotidase III-like protein, or cN-IIIB, has been characterized in human and *Drosophila*. cN-IIIB exhibits a strong substrate preference for the modified nucleotide 7-methylguanosine monophosphate but the structural reason for this preference was unknown. Here, we present crystal structures of cN-IIIB from *Drosophila melanogaster* bound to the reaction products 7-methylguanosine or cytidine. The structural data reveal that the cytosine- and 7-methylguanine moieties of the products are stacked between two aromatic residues in a coplanar but off-centered position. 7-methylguanosine is specifically bound through π-π interactions and distinguished from unmodified guanosine by additional cation-π coulomb interactions between the aromatic side chains and the positively charged 7-methylguanine. Notably, the base is further stabilized by T-shaped edge-to-face stacking of an additional tryptophan packing perpendicularly against the purine ring and forming, together with the other aromates, an aromatic slot. The structural data in combination with site-directed mutagenesis experiments reveal the molecular basis for the broad substrate specificity of cN-IIIB but also explain the substrate preference for 7-methylguanosine monophosphate. Analyzing the substrate specificities of cN-IIIB and the main pyrimidine 5′-nucleotidase cN-IIIA by mutagenesis studies, we show that cN-IIIA dephosphorylates the purine m^7^GMP as well, hence redefining its substrate spectrum. Docking calculations with cN-IIIA and m^7^GMP as well as biochemical data reveal that Asn69 does not generally exclude the turnover of purine substrates thus correcting previous suggestions.

## Introduction

Nucleoside monophosphate phosphohydrolases or 5′-nucleotidases (EC 3.1.3.5) are enzymes catalyzing the hydrolytic dephosphorylation of nucleoside monophosphates to nucleosides and orthophosphate (nucleoside monophosphate+H_2_O→nucleoside+PO_4_^3–^) [1]. As catabolic enzymes, nucleotidases participate in the regulation of nucleotide levels in living cells [2], [3]. In humans, seven different 5′-nucleotidases have been characterized differing with respect to their substrate specificity, subcellular localization, oligomerization state and size [4]. Five of them, namely cytosolic 5′-nucleotidase IA (cN-IA), cytosolic 5′-nucleotidase IB (cN-IB), cytosolic 5′-nucleotidase II (cN-II), cytosolic 5′-nucleotidase IIIA (cN-IIIA; previously called cN-III) and cytosolic 5′(3′)-deoxyribonucleotidase (cdN) are located in the cytosol whereas one is mitochondrial (mitochondrial-5′(3′)-deoxyribonucleotidase, mdN) and one is an extracellular enzyme (ecto-5′-nucleotidase, eN). Crystal structures are available for five of the seven nucleotidases including the mitochondrial (mdN) [5]–[7] and the extracellular nucleotidase (eN) [8], [9] as well as the cytosolic nucleotidases cdN [7], cN-II [10]–[12] and cN-IIIA [13], [14]. Although the intracellular 5′-nucleotidases generally share a low sequence similarity, all enzymes are magnesium-dependent and belong to the haloacid dehalogenase (HAD) superfamily [15]. They are characterized by a modular architecture, containing a structurally conserved HAD core domain, which contains the catalytic residues, and an enzyme-specific cap domain consisting of one or more cap insertions. The various substrate specificities of intracellular 5′-nucleotidases are mainly achieved by different cap domains, which contain the substrate specificity motif S, while the catalytic platform is provided by the conserved HAD core domain [15].

Within the HAD core domain, the canonical motifs I-III include the residues for magnesium ion coordination and catalysis [15]–[17]. During catalysis of cytosolic 5′-nucleotidases, the first aspartate of motif I (DXDX[T/V]) performs a nucleophilic attack on the phosphate moiety of the bound substrate involving a pentavalent phosphate transition state [18]–[20]. Consequently, the phosphoryl group is transferred to the carboxyl group of this aspartate forming a covalent phospho-enzyme intermediate, which is subsequently resolved by hydrolysis. For that purpose, the second aspartate of this motif is thought to coordinate the attacking water molecule [13]. During the reaction, a serine or threonine residue from motif II (ΦΦΦ[S/T]), preceded by 3 hydrophobic Φ-residues, interacts with the phosphate group and stabilizes the pentavalent phosphate transition state. The conserved catalytic motif III (K(X)_x_ D(X)_0–4_ D) contains a lysine and two aspartate residues that stabilize the phosphorylated intermediate and coordinate the magnesium ion, respectively [5]. Although the catalytic mechanism of 5′-nucleotidases has been characterized in detail due to a number of available crystal structures and biochemical data, the structural basis for substrate recognition and discrimination remains elusive for some of them.

Recently, a novel member of the family of cytosolic 5′-nucleotidases, the cytosolic 5′-nucleotidase III-like protein, has been characterized in humans and in *Drosophila* [21] and subsequently renamed cN-IIIB (UniProt IDs Q969T7 and Q9W197). Human cN-IIIB (*^Hs^*cN-IIIB) exhibits a sequence identity and similarity of 59% and 80% to the human cytosolic 5′-nucleotidase IIIA (*^Hs^*cN-IIIA). Interestingly, although both enzymes share significant sequence similarity they appear to have different substrate specificities [21]. *^Hs^*cN-IIIA is the main pyrimidine 5′-nucleotidase in erythrocytes; its activity was thought to be restricted to (d)CMP, (d)UMP and dTMP [22]. A lack of this enzyme in human erythrocytes results in hemolytic anemia, which probably is due to the accumulation of pyrimidine nucleotides and precipitation of unhydrolyzed ribosomal RNA [23]–[25]. In contrast to human cN-IIIA, human cN-IIIB has a broad substrate specificity, efficiently converting CMP and UMP and, with significantly lower efficiency, GMP and AMP [21]. Remarkably, cN-IIIB dephosphorylates the modified purine 7-methylguanosine monophosphate (m^7^GMP) with the lowest *K*_m_ among all substrates tested. Due to the presence of the mRNA cap structure, 7-methylguanosine nucleotides are released during degradation of all eukaryotic mRNAs: Either Dcp2 and related enzymes cleave off m^7^GDP, which is further converted to m^7^GMP, or m^7^GMP is released directly by the scavenger decapping enzyme DcpS [26]–[28]. Additionally, 7-methylguanosine was shown to be present in several tRNA and rRNA species of archaea, bacteria and eukarya [29]. Until recently, it was not known by which enzymatic activity m^7^GMP is further degraded after RNA decay. As a potential alternative to decay, accumulation of m^7^GMP in the cytosol and subsequent incorporation into nucleic acids seems possible but biologically highly undesirable [26], [30]–[32].

To gain insight into the broad substrate specificity of cN-IIIB and especially its unusual preference for m^7^GMP, we determined crystal structures of *Drosophila melanogaster* cN-IIIB (*^Dm^*cN-IIIB) bound to different reaction products. The overall structure of *^Dm^*cN-IIIB is similar to cN-IIIA, however, critical residues conferring substrate specificity and determining the size of the substrate-binding pocket are different. The co-crystallized reaction product, 7-methylguanosine or cytidine, is bound in a deep cavity formed by the cap domain and the HAD core domain and is surrounded by three aromatic amino acids forming an aromatic slot. The structures explain why cN-IIIB prefers m^7^GMP over GMP as substrate but also efficiently converts pyrimidines. In combination with biochemical data from mutagenesis experiments of cN-IIIA and cN-IIIB and molecular docking calculations we define the structural basis for m^7^GMP dephosphorylation by 5′-nucleotidases.

## Results and Discussion

### Crystallization and Structure Determination of *^Dm^*cN-IIIB

*^Dm^*cN-IIIB was crystallized in the presence of the reaction products 7-methylguanosine (m^7^G) or cytidine, and a mixture of AlCl_3_ and NaF. The crystallographic phase problem was solved by means of molecular replacement using the crystal structure of mouse cN-IIIA as starting model [13] (PDB ID 2G08). The structure model of *^Dm^*cN-IIIB bound to m^7^G was refined at a resolution of 1.65 Å to R and R_free_ values of 15.9% and 19.4%, respectively. Diffraction data and refinement statistics are summarized in **Table 1**.

**Table 1:** X-ray diffraction data and structure refinement statistics of *Drosophila melanogaster* cN-IIIB bound to different reaction products.

Crystal	cN-IIIB+m^7^Guanosine+MgF_3_^–^	cN-IIIB+cytidine
*Data collection*		
Space group	*P*2_1_	*P*2_1_
*Cell dimensions*		
a, b, c (Å)	46.67, 99.11, 74.12	46.60, 99.35, 72.81
α, β, γ (°)	90.00, 90.99, 90.00	90.00, 92.46, 90.00
Wavelength (Å)	0.920	0.918
X-ray source	BL14.1, BESSY (Berlin)	BL14.1, BESSY (Berlin)
Resolution range (Å)	46.67–1.65 (1.75–1.65)	35.88–2.05 (2.15–2.05)
No. of reflections	240166 (28865)	112476 (15349)
Completeness (%)	94.5 (81.7)	96.5 (97.1)
R_merge_ (%)	5.3 (54.7)	8.3 (49.2)
Average I/σ	15.5 (2.2)	11.1 (2.4)
Redundancy	3.1 (2.7)	2.8 (2.9)
*Refinement*		
Resolution (Å)	41.19–1.65	35.87–2.05
Molecules per a.u.	2	2
*No. of atoms*		
Protein	4980	4733
Ligands and ions	83	36
Waters	584	297
R_work_ (%)	15.91	22.24*
R_free_ (%)	19.42	25.47*
*Average B factors (Å^2^)*		
Protein	20.35	28.85
Ligands and ions	20.42	31.40
Waters	30.87	28.73
*RMS deviations*		
Bond lengths (Å)	0.012	0.006
Bond angles (°)	1.4	1.0
*Ramachandran statistics (%)*		
Favored	97.4	96.7
Allowed	2.6	3.3
Outliers	0.0	0.0

Values in parentheses indicate the specific values in the particular highest resolution shell.

* Twinned refinement: twin law = h,-k,-l; twin fraction = 0.10.

The final model of the *^Dm^*cN-IIIB m^7^G complex contains two molecules in the asymmetric unit with one continuous segment encompassing residues 13–311 of molecule A as well as residues 13–312 of molecule B, both superposing with a root mean square deviation (rmsd) of 0.86 Å for 288 common C_α_ atoms (residues 13–103 and 115–311). After all amino acids had been placed, additional density was observed in the active center, which, based on its shape, could be assigned as an octahedrally coordinated magnesium ion, the co-crystallized product nucleoside m^7^G as well as a metal fluoride transition state analog. It was shown that for pH values higher than 7.0, enzyme-MgF_3_ complexes rather than enzyme-AlF_3_ complexes are formed [33], [34]. Since both aluminum and magnesium were present during crystallization, we interpreted the density as MgF_3_^–^.

### Overall Structure of *^Dm^*cN-IIIB

**Figure 1:**
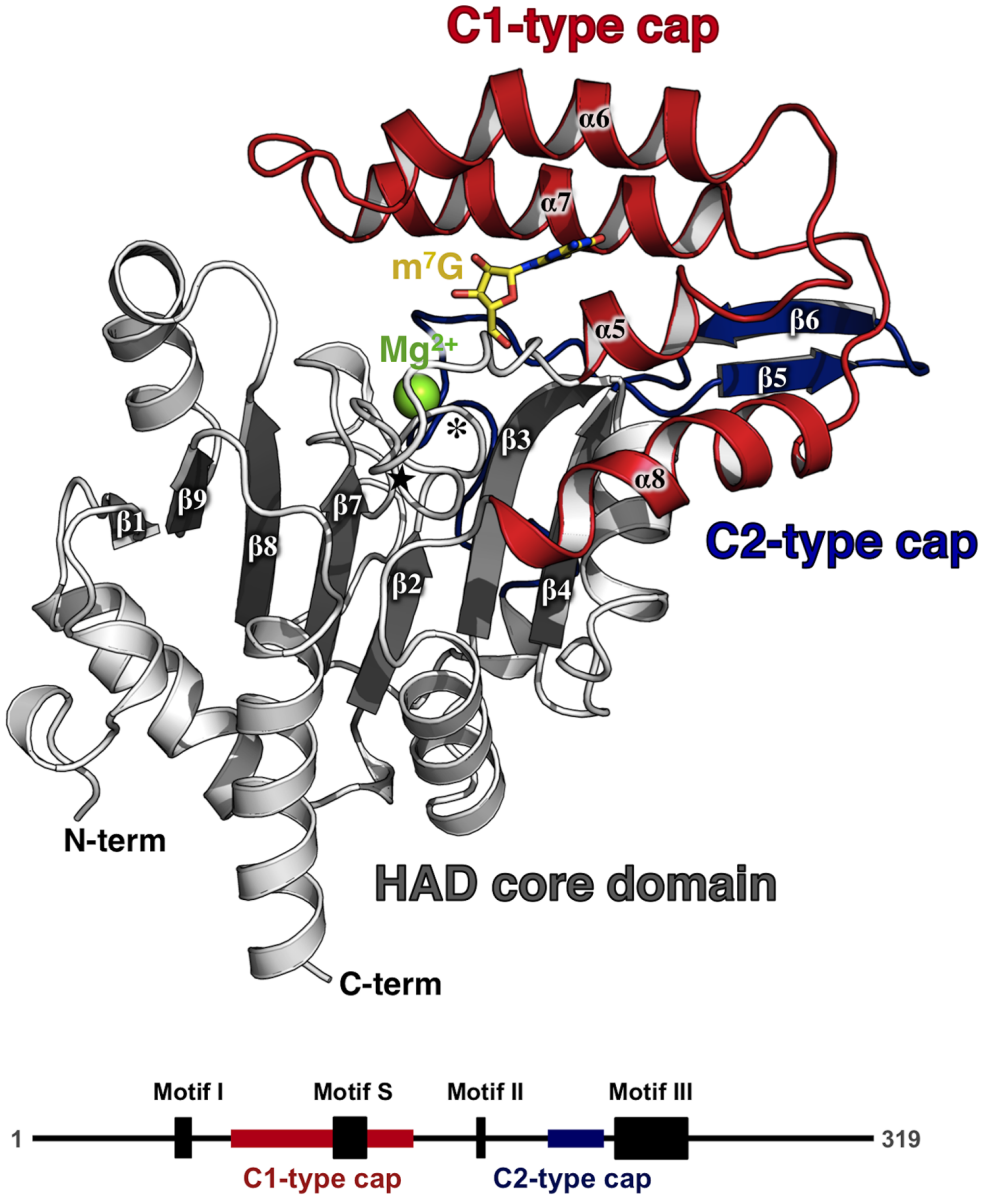
Overall structure of *Drosophila melanogaster* cN-IIIB. *^Dm^*cN-IIIB consists of the conserved central HAD core domain (grey β-strands and white α-helices) and a cap domain. The cap domain packs against the top of the HAD core and is composed of two regions, a C1-type cap (red) and a C2-type cap (blue). The “squiggle” and the “flap”, both structural key features of 5′-nucleotidases, are marked with an asterisk and a black star, respectively. Secondary structure elements as well as termini and the bound magnesium ion (green sphere) are labeled. The co-crystallized reaction product 7-methylguanosine (m^7^G) is bound between the cap domain and the HAD core and is represented as stick model (carbon in yellow, nitrogen in blue, oxygen in red). The lower panel shows a schematic representation of *^Dm^*cN-IIIB with the canonical 5′-nucleotidase motifs I-III and S as well as the positions of the cap insertions highlighted as boxes and labeled.

The overall structure of *^Dm^*cN-IIIB consists of two domains: the globular core domain and a smaller cap domain. The globular core domain is a haloacid dehalogenase domain (HAD domain) and resembles a Rossmannoid-like α/β structure with a central seven-stranded β-sheet exhibiting the topology β1↓β9↑β8↑β7↑β2↑β3↑β4↑ (**Figure 1**). Between two consecutive β-strands, one or more α-helices build right-handed crossover connections generating a β-sheet flanked by several α-helices on either side. The β-sheet is twisted in a way that the two outermost strands are arranged in an almost perpendicular orientation with respect to each other. The HAD core is distinguished from other Rossmannoid folds by two additional structural key features. The “squiggle” (amino acids Phe56-Thr61, **Figure S1**) is a single helical turn (in some structures resembling a π-helix) located immediately downstream of the first core β-strand (β2) and is followed by the “flap” – a short β-turn (amino acids Thr65-Gly68). Both structural motifs provide the structural flexibility required for nucleotidases to switch between open (substrate binding) and closed (catalytically active) conformations. The sequence of the HAD domain in *^Dm^*cN-IIIB includes two insertions, the caps, at two distinct sites. The first one lies between β2 and α-helix 9 and, due to its location, is termed C1-type cap [15]. It comprises the α-helices 5–8 (amino acids 74–143) (**Figure 1**). The second insertion is named C2-type cap, compasses β5 and β6 (amino acids 195–216) and is positioned between β4 and α-helix 11. These two insertions protrude from the α/β Rossmannoid core of the structure and form the cap domain, which serves as a movable lid for the active site of the enzyme as suggested by different orientations of the domain in crystal structures of cN-IIIA [13], [14] and cN-IIIB (reported here). Moreover, it represents the main substrate specificity determinant of cN-IIIB as it harbors critical residues for substrate binding (see below).

### Catalytic Motifs and Active site of *^Dm^*cN-IIIB

**Figure 2:**
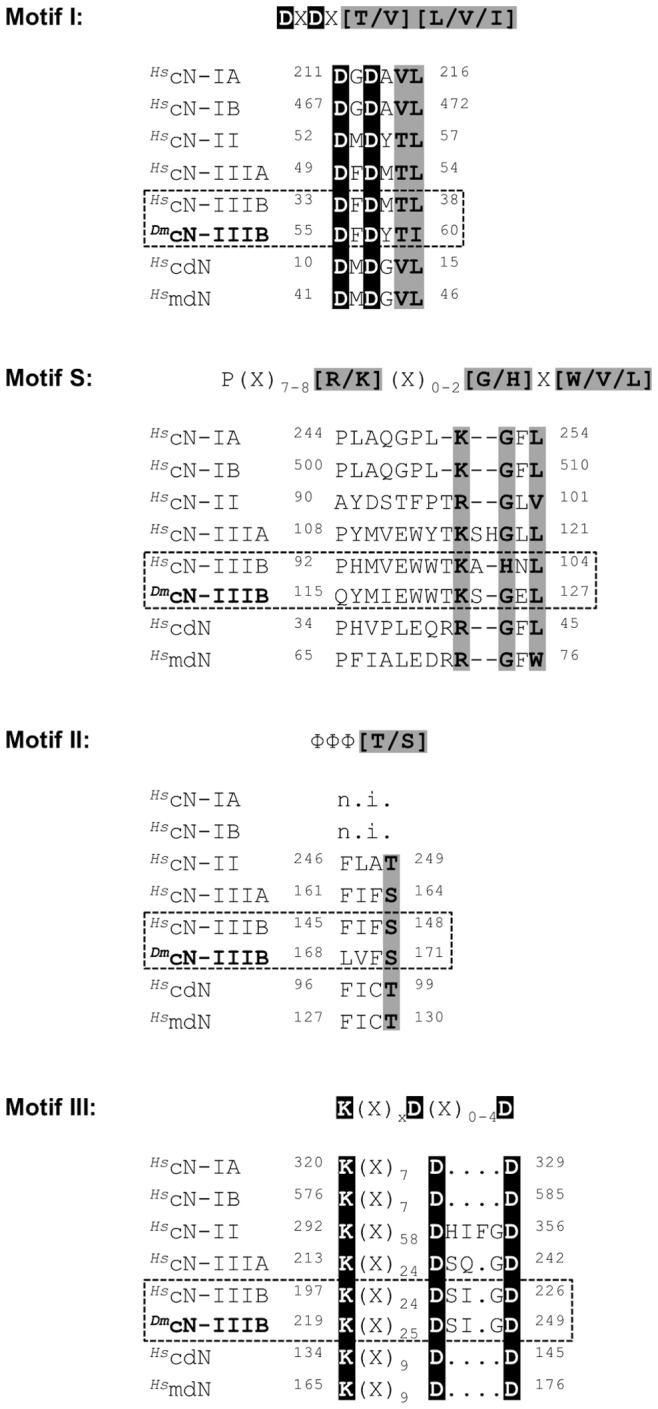
Conserved catalytic sequence motifs of the intracellular human 5′-nucleotidases and cN-IIIB. Sequence conservation of catalytic motifs of 5′-nucleotidases completed by the new member cN-IIIB. The respective motifs of *^Hs^*cN-IIIB and *^Dm^*cN-IIIB have been deduced from the crystal structure and sequence analyses. Shading indicates the different degrees of conservation from absolutely conserved (black) to partly conserved (grey). Motif I, II and III contain conserved catalytic residues and motif S contains residues involved in substrate recognition. Amino acids are numbered and consensus sequences are given above the alignment.

**Figure 3:**
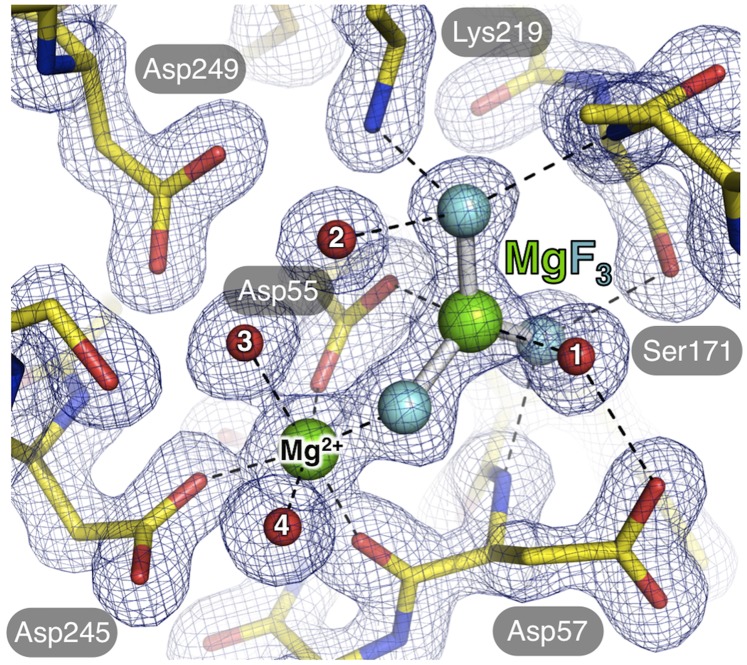
Organization of the catalytic center of *^Dm^*cN-IIIB. The magnesium ion (green sphere) is octahedrally coordinated by several residues of *^Dm^*cN-IIIB shown as stick model (carbon in yellow, nitrogen in blue, oxygen in red) as well as several water molecules (red). One equatorial coordination position of Mg^2+^ is occupied by a fluorine atom of the co-crystallized metal fluoride MgF_3_^–^ (magnesium in green; fluorine in light blue). MgF_3_^–^ is coordinated by Asp55, Asp57, Lys219, Ser171 as well as water molecules. Note that the coordination of MgF_3_^–^ represents the pentavalent phosphate transition state in the nucleotidase reaction cycle. Contacts are represented by dashed lines and the model is defined by a representative 2 mF_o_ – DF_c_ electron density map (blue mesh) contoured at a sigma level of 2.0.

*^Dm^*cN-IIIB contains all canonical sequence motifs typically present in cytosolic 5′-nucleotidases, namely motifs I, II, III as well as motif S. A sequence alignment of the canonical catalytic motifs for all intracellular 5′-nucleotidases completed by the new member cN-IIIB is shown in **Figure 2**. Motif I (^55^ DFDYTI^60^ in *^Dm^*cN-IIIB) is located in the loop connecting β-strand 2 and α-helix 5 (**Figure 1** and **Figure S1**) and contains two aspartate residues separated by a hydrophobic amino acid. In homologous enzymes, the first aspartate was shown to be the nucleophile responsible for the in-line attack on the α-phosphate of the bound nucleoside monophosphate. In the reaction, the cleaved-off phosphate is transferred to this aspartate, initially generating a pentavalent transition state and, later, a covalent phosphoenzyme intermediate. During subsequent hydrolysis of the phosphoenzyme intermediate, the side chain carboxyl of Asp57 is likely to be involved in donation of a proton to the bound phosphate, thus acting as the general acid-base residue in this reaction [13]. In fact, this state is represented in the crystal structures with the transition state analog MgF_3_^–^ coordinated by Asp55 and the attacking water occupying the second apical coordination site (**Figure 3**). The transferred proton may originate from the water molecule, which is further bound by the side chains of Asp57 (motif I) and Ser171 belonging to motif II in the crystal structure (see below). Alternatively, the proton may stem from the 5′-hydroxyl of another bound nucleoside, which results in a phosphotransferase activity observed in some members of the cytosolic 5′-nucleotidase family [22], [35]. Motif II is a four amino acid stretch with the sequence ^168^LVFS ^171^ and is located in β-strand 3 of *^Dm^*cN-IIIB (**Figure 1**). Motif III is of variable length in the different nucleotidases and includes a highly conserved lysine residue as well as two invariant aspartates (^219^ K(X)_25_ DSIGD ^249^ in *^Dm^*cN-IIIB).

In the crystal structure of *^Dm^*cN-IIIB bound to m^7^G, the magnesium ion is octahedrally coordinated by the side chain carboxyls of Asp55 and Asp245 (motif I and III) as well as by a water molecule and a fluorine atom occupying the four equatorial positions (**Figure 3**). The two apical coordination sites are occupied by one water molecule and the main chain carbonyl of Asp57, respectively. The magnesium ion of MgF_3_^–^ makes an apical contact to the remaining oxygen of the Asp55 carboxyl group confirming the predicted role of this amino acid in the reaction (**Figure 3**). The three fluoride ions are in contact with the side chains of Ser171 of motif II, Lys219 of motif III and the magnesium ion, respectively. The position and function of Lys219 of *^Dm^*cN-IIIB is reminiscent of the basic residues termed arginine fingers that stabilize the negative charge on reaction intermediates in many other phosphohydrolases [15]. Finally, the invariant residues Asp245 and Asp249 of motif III coordinate the magnesium ion (see above).

### 7-methylguanosine Binding by *^Dm^*cN-IIIB

It has recently been shown that the Michaelis constant (*K*_m_) for the *^Dm^*cN-IIIB-mediated dephosphorylation of m^7^GMP (*K*_m_ = 13 µM) is significantly lower than for AMP (32 µM), CMP (48 µM), UMP (91 µM) and GMP (102 µM). For the human ortholog *^Hs^*cN-IIIB, the preference for m^7^GMP in terms of the *K*_m_ value is even more pronounced [21]. At least to some extent, this also reflects a higher binding affinity for m^7^GMP compared to the unmodified purines and pyrimidines. However, the structural basis for this preference, unusual among 5′-nucleotidases, and the broad substrate specificity in general has so far been unknown.

**Figure 4:**
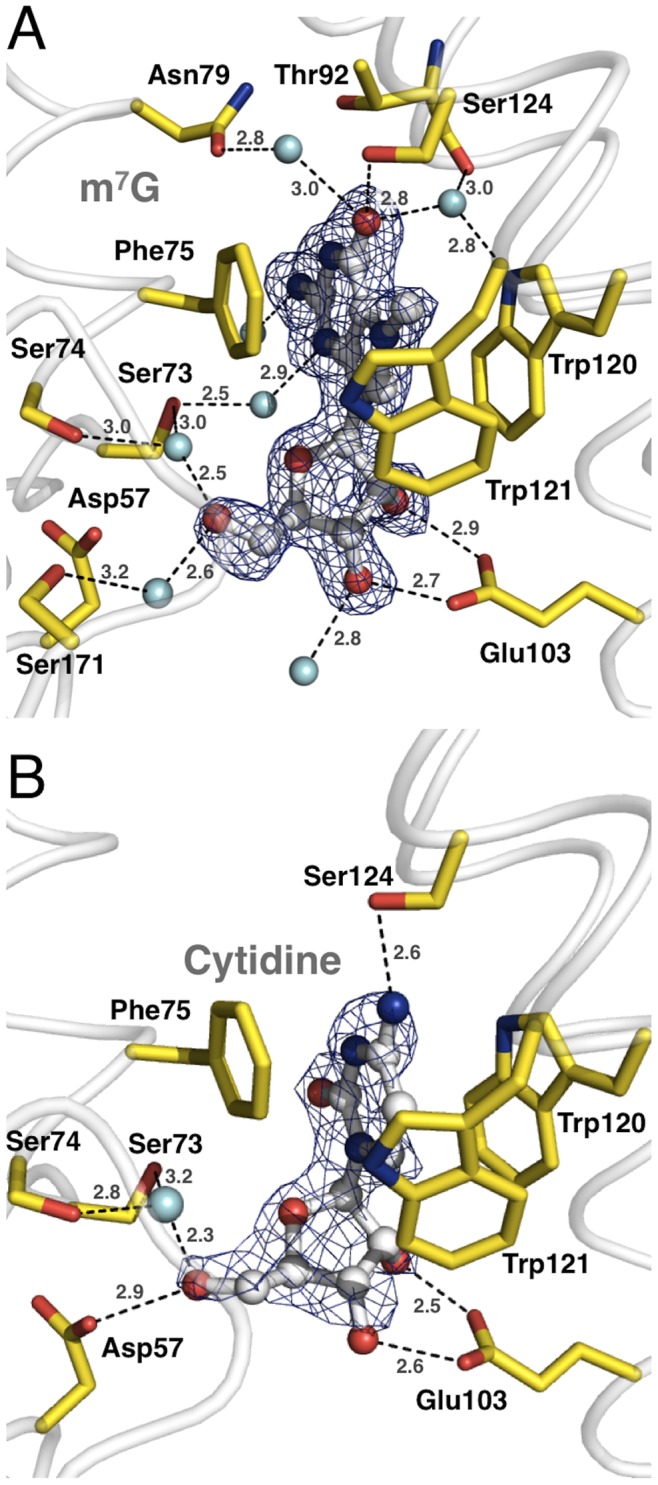
Detail view on the substrate-binding pocket of *^Dm^*cN-IIIB. (A) The co-crystallized reaction product 7-methylguanosine is shown in ball-and-stick mode (carbon in grey, nitrogen in blue, oxygen in red) and is defined by an mF_o_ – DF_c_ omit-electron density map (blue mesh) contoured at a sigma level of 3.0. m^7^G is stacked in a coplanar but off-centered fashion by Phe75 and Trp120 (colors as in **Figure 3**). Trp121 packs by T-shaped edge-to-face stacking against the bound product and binding is additionally supported by numerous direct or water-mediated hydrogen bonds (dashed lines with distances) to various protein residues of *^Dm^*cN-IIIB. (B) Binding of the reaction product cytidine by *^Dm^*cN-IIIB. In contrast to m^7^G, the nucleobase moiety of cytidine makes only a hydrogen bond to Ser124.

**Figure 5:**
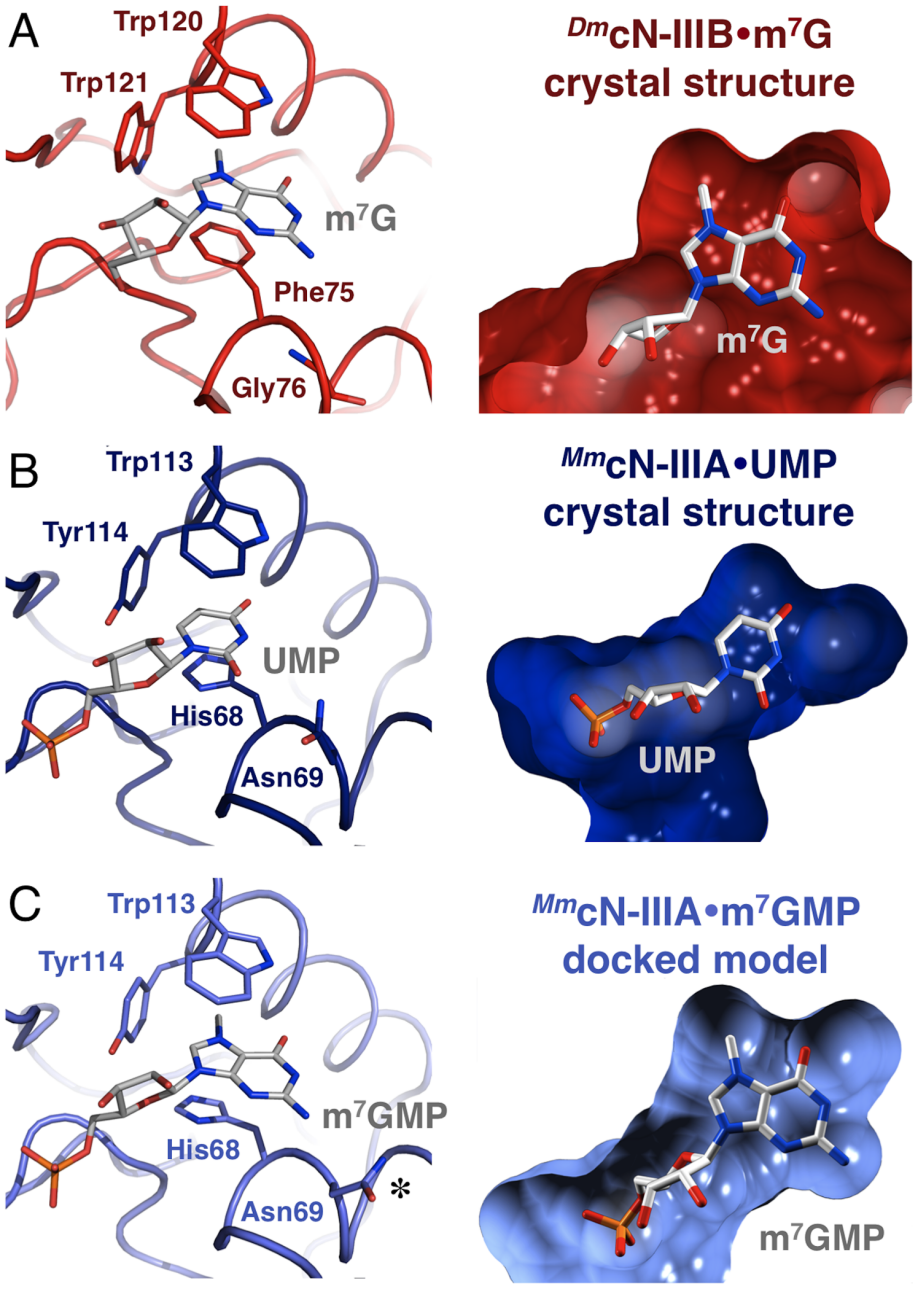
Structural comparison of *^Dm^*cN-IIIB and *^Mm^*cN-IIIA. Detail view on the substrate-binding pockets of *^Dm^*cN-IIIB bound to m^7^guanosine in red (A) and *^Mm^*cN-IIIA bound to UMP in dark blue (B). The left panel shows the binding pocket with labeled residues and substrates represented as sticks. Note that Phe75 of *^Dm^*cN-IIIB is replaced by a histidine in *^Hs^*cN-IIIA while the Trp121 of *^Dm^*cN-IIIB is replaced by a tyrosine in *^Hs^*cN-IIIA. In the right panel the substrate-binding pockets are shown as surfaces, which were calculated using a probe radius of 1.65 Å and for clarity reasons were clipped at the level of the nucleobases. Note that the substrate-binding pocket for m^7^G (cN-IIIB, red surface) is significantly larger than the UMP binding pocket of cN-IIIA (blue surface), which was thought to be the structural reason for its purine substrate exclusion. (C) Orientation of substrate binding residues and the corresponding pocket after docking m^7^GMP to *^Mm^*cN-IIIA (light blue). Note that Asn69 (marked by an asterisk) rotates by 180 degrees in comparison to (B) and adopts another common rotamer thus enlarging the binding pocket and neatly accommodating the m^7^GMP.

Overall, the C1-type cap containing the substrate specificity motif (motif S) together with the HAD core domain generates a substrate-binding pocket which neatly accommodates the bound 7-methylguanosine in a deep binding cleft (**Figure 1**). Both the base and the ribose moiety of m^7^G are in contact with diverse protein residues. In detail, the 7-methylguanine moiety of the nucleoside is stacked in a parallel displaced orientation between the aromatic side chains of Trp120 and Phe75, bridging typical stacking distances of 3.5 Å between the side chains and the base (**Figure 4A**). Besides a π-π stacking interaction resulting from the parallel orientation of the π-electron systems of the purine ring and the aromatic amino acid side chains, there is also a cation-π stacking coulomb interaction between the side chain π-electrons of Trp120 or Phe75 and the positive charge of m^7^G. It has been shown that the positive charge of m^7^G mainly localizes to nitrogen 7 [36], which interestingly is positioned above the center of the indole ring of the stacking Trp120. The cation-π stacking thus seems to be a major contribution and one of the structural reasons for the high affinity of *^Dm^*cN-IIIB for the monomethylated m^7^GMP and the discrimination against the unmethylated GMP. Notably, the binding pocket is completed by another aromatic side chain of Trp121, which packs by T-shaped edge-to-face stacking perpendicular against the methylated purine ring, bridging a distance of 3.8 Å between the methyl group of m^7^G and the plane of Trp121. This binding pattern results in the formation of an aromatic slot by Trp120, Phe75 and Trp121 neatly enclosing the base and shielding it from surrounding charged amino acids and water molecules (**Figure 4A** and **5A**). This binding mode is reminiscent of 5′-cap dinucleotide binding by the nuclear import adapter Snurportin 1 [37], where the hypermethylated 5′-guanine of the snRNA is stacked between a tryptophan side chain and the second RNA base while it is shielded from the aqueous surrounding by a second tryptophan. Moreover, in *^Dm^*cN-IIIB the residues Ser124, Phe209, Trp120, Trp121 and Phe75 form a cavity that harbors the N7-methyl group of the modified base (**Figure 5A**). In addition, residues Phe78, Thr128, Leu199, Phe209 and Leu174 form a deep and elongated second cavity near O6 of the m^7^G, which would be able to accommodate diverse alkylated adenine nucleotides such as N6-isopentyladenosine, N6-glycinylcarbamoyladenosine or N6-N6-dimethyladenosine that have been found in different RNA species [29]. Thus, cN-IIIB may not only convert m^7^GMP but also a number of additional modified or alkylated nucleotides derived from the degradation of various RNA species.

In addition to the base, the 2′ and 3′ hydroxyls of the ribose make polar contacts to the carboxyl group of Glu103 (**Figure 4A**). The ribose 5′ hydroxyl makes contacts with the side chain of Asp57 as well as two water molecules, one of which would be in the position for the in-line attack on the phospho-aspartate 55 (see above). Moreover, O6 of the purine ring is in close contact with Ser124 of motif S as well as with two conserved water molecules. Furthermore, nitrogens N1, N2 and N3 of the base are in contact with several water molecules coordinated by protein residues.

In summary, m^7^GMP is converted with a ∼10-fold lower *K*_m_ compared to GMP for *^Dm^*cN-IIIB and with a 45-fold lower *K*_m_ for the human ortholog. The different *K*_m_ values for GMP and m^7^GMP underline the importance of the contribution of the cation-π interaction between the methylated base and the aromatic side chains. Moreover, the slightly higher affinity of human cN-IIIB for m^7^GMP compared to *^Dm^*cN-IIIB could be explained by the fact that one of the stacking residues in the *Drosophila* protein, Phe75, is replaced by a tyrosine (Tyr60) in the human counterpart (**Figure S1**). It has been shown for a viral m^7^G-cap binding methyltransferase (VP39) that the exchange of such a stacking tyrosine in the binding pocket by phenylalanine reduces the affinity for the m^7^G-cap by a factor of four [38]. Whereas the human enzyme, compared to *^Dm^*cN-IIIB, has a 2-fold higher apparent affinity for m^7^G, it shows reduced affinities for the substrates CMP (2-fold higher *K*_m_), GMP (4-fold higher *K*_m_), AMP (14-fold higher *K*_m_) and UMP (5-fold higher *K*_m_). This may, at least in part, be a result of hydrogen bonding between the nucleobase and Ser124 of *^Dm^*cN-IIIB; this serine residue is replaced by an alanine in the human protein.

### Cytidine Binding by *^Dm^*cN-IIIB

In contrast to other cytosolic 5′-nucleotidases, cN-IIIB dephosphorylates both pyrimidines and purines (see above). To compare binding of purines and pyrimidines and explain this broad substrate specificity, we crystallized *^Dm^*cN-IIIB in complex with cytidine and solved the crystal structure by means of molecular replacement using the structure of the *^Dm^*cN-IIIB m^7^G complex as starting model. Remarkably, there was no density for MgF_3_^–^ in the active site of the cytidine bound structure. The overall conformation of *^Dm^*cN-IIIB in complex with cytidine is highly similar to the m^7^G-bound structure with an rmsd of 0.32 Å for 293 common C_α_ atoms of the molecules A. Differences between both structures are mainly restricted to the flap (amino acid Thr65-Gly68), which is not defined in the cytidine bound structure and thus most likely disordered. In contrast to the m^7^G-bound structure, several amino acids in the C1-type cap have poorly defined side chains, however all main chain atoms as well as the important residues for substrate binding are well defined in the electron density map.

*^Dm^*cN-IIIB binds cytidine in a similar fashion as m^7^G, using the same substrate-binding pocket, however the hydrogen-bonding pattern is different (**Figure 4B**). The pyrimidine ring is stacked in an almost coplanar fashion between Phe75 and Trp120, bridging plane distances of 3.5 and 3.4 Å, respectively. The side chain of Trp121 packs in an edge-to-face fashion with a distance of 3.5 Å to the C5 atom of cytidine. When superimposed to m^7^G, the centroid of the pyrimidine ring is in close proximity to the methylated nitrogen 7 of the guanine base, thus the cytosine ring captures the position of the 5-membered imidazole ring of m^7^G.

Besides the described stacking interactions, the cytidine makes only one direct hydrogen bond to Ser124, which is in contact to the N4 of the base (**Figure 4B**). Consequently, the water-mediated interactions of Asn79, Thr92, Trp120 and Ser171 of *^Dm^*cN-IIIB bound to m^7^G are missing in the cytidine-bound structure, partly explaining the lower affinity of the enzyme for pyrimidines.

### Comparison of Open and Closed States of cN-IIIB and cN-IIIA

**Figure 6:**
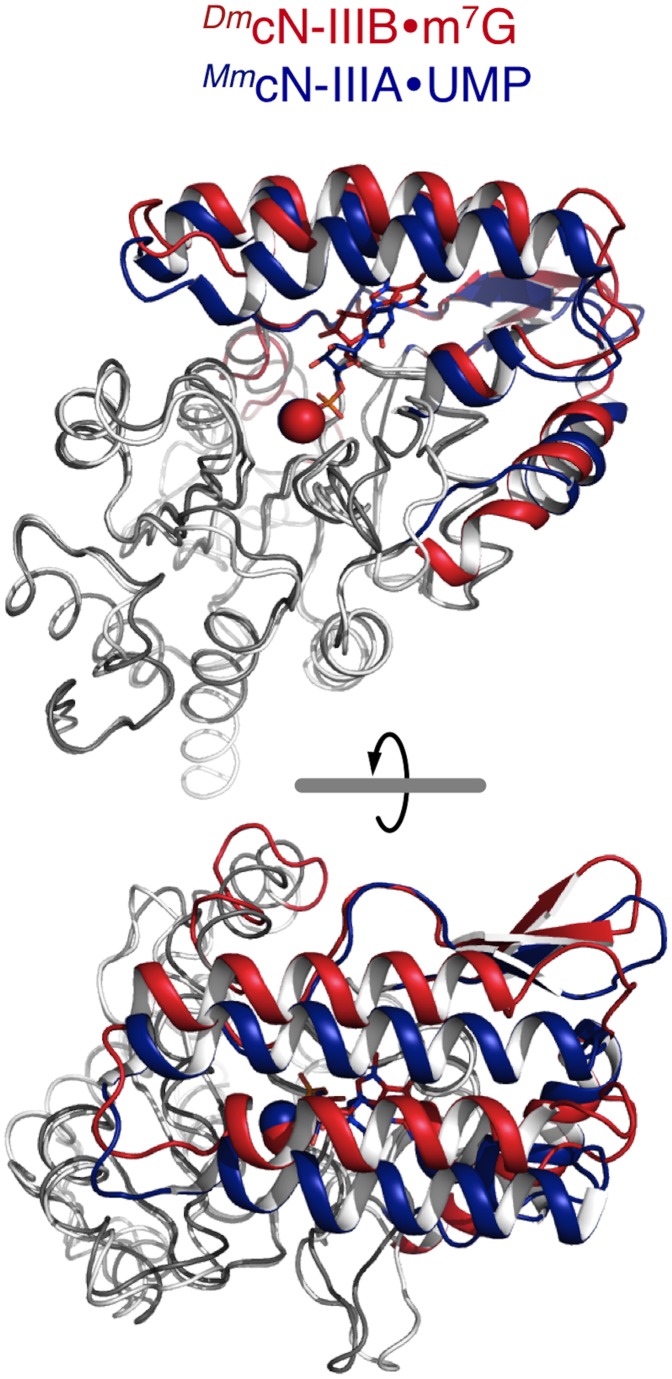
Overall structural comparison of *^Dm^*cN-IIIB bound to m^7^G with *^Mm^*cN-IIIA bound to UMP. Superposition of *^Dm^*cN-IIIB (HAD core in white; cap domain in red) and cN-IIIA (HAD core in grey; cap domain in blue) showing the overall structural similarity of both enzymes. Note that the cap domain of cN-IIIB is shifted by 4 Å when the structures are superposed *via* their the HAD core. Thus, the substrate-bound cN-IIIA adopts a closed conformation while the product-bound cN-IIIB represents an open conformation. A movie of the trajectory from the open-to-closed state is shown in **Movie S1**.

Although all cytosolic 5′-nucleotidases share similar HAD core folds, overall the structures are different due to the structural variability of their cap domains, which confer substrate specificity (**Figure S3**). The modular architecture of nucleotidases, with the conserved HAD core domain as catalytic platform and the structurally variable cap domain, allows different substrate specificities while maintaining catalytic efficiency. The closest nucleotidase homolog to cN-IIIB is the cytosolic 5′-nucleotidase IIIA (former cN-III, sequence alignment in **Figure S2**), which has been structurally and functionally characterized in detail [13], [14]. *^Hs^*cN-IIIA (originally called pyrimidine-specific 5′-nucleotidase 1) catalyzes the hydrolysis of CMP and UMP [25]. Several crystal structures of cN-IIIA in complex with the substrate UMP [14] or representing different states of the reaction mechanism [10], [13] have been solved showing the molecular basis for substrate binding and catalysis. Superposition of the HAD core domains of cN-IIIA and cN-IIIB shows that the orientation of the cap domains with respect to the HAD core are slightly different (**Figure 6**). The crystal structures of the cN-IIIA UMP complex and the cN-IIIB m^7^G complex superpose with an rmsd of 1.19 Å for 218 common C_α_ atoms. In the crystal structure of the cN-IIIA UMP complex, the enzyme completely encloses the substrate in a deep cavity between the HAD core and the cap domain [14]. In contrast, in cN-IIIB bound to m^7^G, the tip of the cap domain (loop between α-helices 6 and 7) is shifted by 4 Å away from the HAD core representing a more open state (**Figure 6** and **Movie S1**). This structural difference in the orientation of the cap domain relative to the HAD core may be due to the fact that cN-IIIA is bound to the reaction substrate including the phosphate (UMP), while cN-IIIB is bound to a reaction product missing the phosphate (7-methylguanosine). Notably, there are several direct contacts of the UMP phosphate with Lys213, Ser164 and Asp51 of the HAD core domain as well as with the active site magnesium ion, which stabilize the closed conformation of cN-IIIA. Instead, the corresponding residues Ser171, Lys219 and Asp55 in cN-IIIB are in direct contact with the transition state analog MgF_3_^–^, which mimics the phosphate moiety already transferred to Asp55 (**Figure 3**). As a consequence, bond cleavage between the α-phosphate, coordinated in part by the HAD core, and the nucleoside, which is tightly bound by the cap domain, allows both domains to move away from each other and to subsequently release the reaction products (**Movie S1**).

### Substrate Specificities of cN-IIIA and cN-IIIB

On the basis of structural data and mutagenesis experiments, it was suggested that the substrate-binding pocket of cN-IIIA is too small to accommodate purines and Asn69 would sterically clash with such substrates (**Figure 5B**), thus limiting the substrate spectrum of cN-IIIA to pyrimidine nucleotides [14]. To characterize the different substrate specificities of cN-IIIA and cN-IIIB and to explain them by structural data we performed mutagenesis studies and activity assays. Our preliminary experiments with a SUMO-tagged version of *^Hs^*cN-IIIA were hampered by solubility problems, but suggested that m^7^GMP could be dephosphorylated, although with a much lower efficiency compared to CMP [21]. With a modified purification procedure, we have now been able to obtain soluble untagged full-length *^Hs^*cN-IIIA, and steady-state kinetic parameters for the dephosphorylation of CMP and m^7^GMP could be determined (**Table 2**). While CMP was the preferred substrate, m^7^GMP was converted with a surprisingly high efficiency, with *k*_cat_/*K*_m_ only 6-fold lower than the specificity constant for CMP. The *K*_m_ for m^7^GMP was about 5-fold lower than for CMP and only about 2-fold higher than the *K*_m_ of *^Hs^*cN-IIIB. Overall, the substrate specificities of *^Hs^*cN-IIIA and -B are more similar than previously thought, especially with respect to the efficient conversion of m^7^GMP by both enzymes.

**Table 2:** Kinetic properties of the cytosolic 5′-nucleotidases IIIA and IIIB.

Substrate	Protein	*K*_m_ (µM)	*k*_cat_ (s^−1^)	*k*_cat_/*K*_m_ (s^−1^ M^−1^)
CMP	*^Dm^*cN-IIIB	48	12	242000
	*^Hs^*cN-IIIA	80	9	118600
	*^Hs^*cN-IIIB	79	7	87100
m^7^GMP	*^Dm^*cN-IIIB	12	4.8	403300
	*^Hs^*cN-IIIA	15	0.3	21100
	*^Hs^*cN-IIIB	8	0.5	64500

The ability of *^Hs^*cN-IIIA to dephosphorylate m^7^GMP with high efficiency is inconsistent with the proposed role of Asn69 in preventing binding of purine nucleotides [14]. This proposal was further tested by mutagenesis studies. For the enzyme variants, the following code was used: Amino acids Phe75-Gly76-Trp120-Trp121 of *^Dm^*cN-IIIB, forming the binding pocket for the nucleobase, are abbreviated FGWW (**Figure 5**). Asn69 in *^Hs^*cN-IIIA corresponds to Gly76 in *^Dm^*cN-IIIB, and the amino acids of *^Hs^*cN-IIIA corresponding to the FGWW motif were abbreviated HNWY (His68-Asn69-Trp113-Tyr114). When Gly76 of the *Drosophila* enzyme was replaced by an asparagine (variant FNWW), the specificity constants for CMP and m^7^GMP changed very little, but the apparent affinity for m^7^GMP was increased rather than decreased (**Table 3**). Introduction of the entire HNWY motif into *^Dm^*cN-IIIB had modest effects, partially converting the substrate preferences of the *Drosophila* enzyme to those of its human counterpart. When, in this context, Asn was replaced by Gly (variant HGWY), the *K*_m_ for both CMP and m^7^GMP was decreased and *k*_cat_/*K*_m_ was strongly increased only for m^7^GMP due to different effects on *k*_cat_ (**Table 3**). In a comparison of FNWY and FGWY, the glycine variant lowered *K*_m_ and increased *k*_cat_ for both substrates, leading to a preference for m^7^GMP (**Table 3**). Thus, the effect of the amino acid occupying the position corresponding to *^Hs^*cN-IIIA Asn69 is context-dependent; a general function of this asparagine in this position to exclude purine substrates is not supported by the data.

**Table 3:** Kinetic properties of the cytosolic 5′-nucleotidases IIIA, IIIB and variants of *^Dm^*cN-IIIB.

Substrate	Protein	*K*_m_ (µM)	*k*_cat_ (s^−1^)	*k*_cat_/*K*_m_ (s^−1^ M^−1^)
CMP	FGWW (*^Dm^*cN-IIIB)	48	12	242000
	FNWW	51	8	166300
	HGWW	490	8	16000
	FGWY	14	6	410200
	HGWY	93	3	32500
	FNWY	70	5	77800
	HNWY	315	9	28800
	HNWY (*^Hs^*cN-IIIA)	80	9	118600
m^7^GMP	FGWW (*^Dm^*cN-IIIB)	12	4.8	403300
	FNWW	2	1.1	545300
	HGWW	40	4.2	105800
	FGWY	≤1	1.9	≥1874000
	HGWY	2	1.5	755400
	FNWY	8	0.3	37800
	HNWY	11	0.5	49500
	HNWY (*^Hs^*cN-IIIA)	15	0.5	21100

To further test the hypothesis that Asn69 of cN-IIIA does not discriminate against purine substrates and explain the ability of cN-IIIA to convert m^7^GMP, molecular docking calculations were performed. The crystal structure of *Mus musculus* cN-IIIA (PDB ID 4FE3) was used to dock the potential substrate m^7^GMP as well as UMP into the “side-chain flexible” substrate-binding pocket, while UMP served as a control of the docking procedure since it is the co-crystallized substrate (see Materials and Methods for details). As expected, docking of UMP to *^Mm^*cN-IIIA led to a model that was virtually identical to the one revealed by the complex crystal structure. It had been suggested previously that Asn69 of cN-IIIA prevents binding of purine substrates for sterical reasons because it restricts the size of the substrate-binding pocket. However, when m^7^GMP was docked into the substrate-binding pocket of cN-IIIA, the side chain of Asn69 rotated by 180 degrees, adopting the conformation of another common rotamer (**Figure 5C**). Due to this movement, the size of the binding pocket was significantly increased, neatly accommodating the purine ring of m^7^GMP. Taken together, the results from activity assays and the docking analyses strongly suggest that Asn69 of cN-IIIA is not discriminating against purine nucleotides.

A second important residue in the substrate-binding pocket of cN-IIIB is Phe75, which is one of the aromates stacking against the nucleobase. Phe75 is replaced by a histidine (His68) in cN-IIIA decreasing the contribution of the π-π stacking interaction significantly although this position is occupied by a tyrosine or histidine in cN-IIIB orthologs of other species as well. Our set of mutants contains three pairs differing in the presence of phenylalanine or histidine at this position: FGWW versus HGWW; FGWY versus HGWY; and FNWY versus HNWY. In all three pairs, and with both CMP and m^7^GMP as substrates, the apparent affinities were consistently higher with the variants containing a phenylalanine at this position (**Table 3**). This fact underlines the importance of π-π stacking in substrate binding by 5′-nucleotidases and may explain in part the generally lower affinities of cN-IIIA for its substrates.

## Materials and Methods

### Protein Expression and Purification for Crystallization

The cDNA of *Drosophila melanogaster* cytosolic 5′-nucleotidase IIIB (*^Dm^*cN-IIIB) (CG3362 on www.flybase.org) encoding the full-length protein (amino acids 1–319) was cloned into the pET-SUMOadapt vector as described [21]. The SUMO-*^Dm^*cN-IIIB fusion construct was expressed in *Escherichia coli* Rosetta 2(DE3) (Merck) at 24°C in kanamycin- and chloramphenicol-containing 2YT-medium. Expression was induced at an OD_600_ of 1.0 by addition of 0.6 mM IPTG. The cells were harvested after 4 hours of induction (5,000×g, 20 min, 4°C) and resuspended in lysis buffer (300 mM NaCl, 50 mM Tris/HCl pH 7.5, 10 mM imidazole, 5 mM MgCl_2_ and 2 mM β-mercaptoethanol). Cells were disrupted using a microfluidizer 110S (Microfluidics) and the lysate, which was clarified by centrifugation (30,000×g, 30 min, 4°C), was loaded onto a HisTrap column (GE Healthcare) equilibrated with lysis buffer. The column was washed with 2 column volumes (CV) of lysis buffer and eluted with a linear gradient of lysis buffer containing 500 mM imidazole. Afterwards, imidazole was removed from the solution by passing the eluate over a 50 ml desalting column (GE Healthcare) equilibrated in desalting buffer (300 mM NaCl, 50 mM Tris/HCl pH 7.5, 5 mM MgCl_2_ and 2 mM β-mercaptoethanol). For cleavage, the SUMO-*^Dm^*cN-IIIB fusion-protein was incubated with SUMO protease (Invitrogen) overnight at 4°C and in a 1∶ 100 molar ratio of protease : fusion protein. In order to remove the His_6_-SUMO tag as well as the protease, the solution was passed through a second HisTrap column. The flow through of this column, mainly containing *^Dm^*cN-IIIB, was finally purified using a Superdex S75 (26/60) gel filtration column (GE Healthcare) in a buffer containing 150 mM NaCl, 20 mM Tris/HCl pH 7.5, 5 mM MgCl_2_ and 2 mM β-mercaptoethanol. The pure protein was concentrated to 15 mg/ml using Millipore concentrators with a molecular weight cut-off of 10,000 Da (Merck) and aliquots were frozen in liquid nitrogen and stored at −80°C.

### Crystallization and Structure Determination

Full-length *^Dm^*cN-IIIB (amino acids 1–319) was crystallized by vapor diffusion in 24-well sitting drop crystallization plates at 20°C. Plate-shaped crystals were obtained in a condition containing 26% (w/v) PEG 4,000, 0.1 M HEPES/NaOH pH 7.5 and 0.2 M NaCl at a protein concentration of 12 mg/ml and when the protein was previously mixed with 5 mM AlCl_3_, 30 mM NaF and with a 15-fold molar excess of m^7^G or cytidine, respectively. The crystals belong to space group *P*2_1_ and were flash cooled in liquid nitrogen after soaking in reservoir solution containing additionally 8% (w/v) PEG 4,000 as cryo protectant. Diffraction images from crystals containing m^7^G or cytidine were collected at the beamline BL14.1 of the Berliner Elektronenspeicherring-Gesellschaft für Synchrotronstrahlung (BESSY II, Berlin) [39]. The datasets were integrated, scaled and merged using *XDS* and *XSCALE* [40], respectively. The structure of the *^Dm^*cN-IIIB m^7^G complex was solved by means of molecular replacement using *MOLREP* [41] as implemented in *CCP4* [42] and the crystal structure of mouse pyrimidine 5′-nucleotidase type 1 (P5N-1 =  cN-IIIA, PDB ID 2G08) as starting model [13]. Full-length mouse cN-IIIA and *^Dm^*cN-IIIB possess a sequence identity and similarity of 33% and 54%, respectively. Atomic coordinates of magnesium trifluoride as well as solvent molecules and the co-crystallized product 7-methylguanosine were added to the model, which was refined by iterative cycles of *PHENIX* [43] and manual building in *Coot* [44] to R and R_free_ values of 15.91% and 19.42%. The crystal structure of the *^Dm^*cN-IIIB cytidine complex was solved by molecular replacement (*MOLREP*) using the previously solved *^Dm^*cN-IIIB m^7^G complex structure as starting model but with the nucleoside and MgF_3_^–^ omitted. Interestingly, there was no density for MgF_3_^–^ in this crystal structure, thus it was not modeled. Atomic coordinates of the cytidine were added to the model and the structure was refined (*PHENIX*) to R and R_free_ values of 22.24% and 25.47% (see **Table 1**).

Structure figures were generated using PyMOL (DeLano, W.L. The PyMOL Molecular Graphics System (2002), DeLanoScientific, USA) or Chimera 1.8 [45].

### Site-directed Mutagenesis

The expression plasmid coding for *^Dm^*cN-IIIB [21] was mutated by site-directed mutagenesis (Stratagene QuikChange protocol) with specific oligonucleotides listed in **Table S1**. Mutations were verified by sequencing and by MALDI-TOF mass-spectrometric analysis of the purified proteins.

### Purification of Proteins for Enzyme Assays

The expression clone containing the cDNA of *^Hs^*cN-IIIA (transcript variant 1; NM_001002010.2) in a pET-SUMOadapt expression vector [46] has been described [21]. *Escherichia coli* Rosetta 2(DE3) cells (Novagen) were transformed with the plasmid and cultured in 800 ml TB medium [47] including kanamycin (30 µg/ml) at 37°C to an OD_600_ of 0.9. Expression was induced by mixing the culture with 800 ml ice-cold TB medium including kanamycin (30 µg/ml) and IPTG (1 mM), and incubation was continued at 16°C for 5 hours. After harvesting, cells were resuspended in 25 ml lysis buffer (20 mM potassium phosphate buffer pH 7.6, 20 mM imidazole, 500 mM KCl, 2 mM MgCl_2_, 5% (w/v) glycerol) and incubated for 1 h at 8°C with 10 mg of lysozyme, 5 mg of DNase I and 30 mg PMSF. The cells were disrupted twice with a French Press and centrifuged at 30,000×g for 75 minutes. The supernatant was loaded onto a 2 ml Ni^2+^-NTA-agarose column (Qiagen) equilibrated in lysis buffer. After washing with lysis buffer, including a step with lysis buffer containing 2 M KCl, bound protein was eluted stepwise with lysis buffer containing increasing concentrations of imidazole (50–500 mM). Fractions containing His_6_-SUMO-cN-IIIA were identified by SDS-PAGE analysis and pooled. For SUMO-cleavage, 1.5 mg recombinant Ulp1 protease (expression plasmid obtained from Christopher D. Lima [48]), 5 mM DTT and 1 mM EDTA were added, and the mixture was dialysed for 4 h at 0°C against lysis buffer containing 10% (w/v) sucrose instead of glycerol. Cleaved protein was again applied to a Ni^2+^-NTA-agarose column, and the flow-through fraction was loaded onto a Heparin column (GE Healthcare) equilibrated in buffer A (20 mM potassium phosphate buffer pH 7.6, 75 mM KCl, 10% (w/v) sucrose). Bound proteins were eluted with 7 CV of a linear gradient from buffer A to buffer B (as buffer A, but containing 2 M KCl). Fractions containing *^Hs^*cN-IIIA were identified by SDS-PAGE analysis and pooled for dialysis against 20 mM HEPES pH 7.6, 200 mM potassium acetate, 3 mM magnesium acetate and 10% (w/v) sucrose. Aliquots were frozen in liquid nitrogen and stored at –80°C. Protein concentration was determined by densitometry of a coomassie-stained SDS-polyacrylamide gel compared to a BSA calibration curve.

*^Dm^*cN-IIIB variants were purified as described [21].

### Biochemical Analysis and Colorimetric Activity Assay of cN-IIIB and cN-IIIA

Colorimetric nucleotidase assays were performed as reported [21]. Substrates were from Jena Bioscience (m^7^GMP) and Sigma Aldrich (CMP). Substrate concentrations were varied between 1 µM and 2 mM. Human enzymes were assayed at 37°C, *Drosophila* enzymes at 25°C. In the case of *^Hs^*cN-IIIA and *^Hs^*cN-IIIB, enzymes were preincubated with 5 mM DTT for 5 minutes at room temperature. The reaction was started by enzyme addition and stopped by mixing an aliquot (2.5–100 µl per time point) with malachit green oxalate/ammonium molybdate reagent. A calibration curve with phosphate concentrations from 1–30 µM was used to estimate orthophosphate released in the enzymatic reaction. Enzymatic steady state parameters were determined as described [21]. The measurements were repeated twice, with kinetic constants varying by up to ±22%. R-values for the hyperbolic Michaelis-Menten fit ranged from 0.9703–0.9962 with 8–16 points per fit.

### Molecular Docking Calculations

Protein and ligand preparation was done using *AutoDockTools 1.5.6* [49] and molecular docking studies were carried out with *AutoDock Vina* [50]. The substrates m^7^GMP and UMP were docked to the active site of the crystal structure of *Mus musculus* cN-IIIA (PDB ID 4FE3). Water molecules and the substrate UMP were omitted and polar hydrogens as well as Gastiger charges were added prior to docking calculations for both docked molecules and the receptor. The charge of the active site magnesium ion was set to +1.2. Calculations were done using a box size of 18×22×22 Å encompassing the ligand binding site as well as all residues that were kept flexible during docking (Thr66, His68, Asn69, Lys89, Trp113, Tyr114, Ser117). Exhaustiveness and energy range of the calculations were set to 1000 and 4, respectively, and docking calculations were repeated twice in order to verify reproducibility of results.

### Accession Numbers

Coordinates and structure factors of the crystal structures of *^Dm^*cN-IIIB bound to 7-methylguanosine or cytidine have been deposited within the protein data bank (PDB) with the PDB IDs 4NV0 and 4NWI, respectively.

## Supporting Information

Figure S1 **Multiple sequence alignment of cytosolic 5′-nucleotidase IIIB from** ***Drosophila melanogaster*** **,** ***Xenopus laevis*** **,** ***Gallus gallus*** **,** ***Mus musculus*** **,** ***Rattus norvegicus*** **and** ***Homo sapiens*** **.** Identical residues are shown on red background, while similar ones are represented in red on white background. Secondary structure elements from the crystal structure of *^Dm^*cN-IIIB are shown above the alignment. Note that important residues from motifs I-III (marked with a black triangle) as well as both tryptophans necessary for substrate binding in *^Dm^*cN-IIIB are strictly conserved among all species while the second stacking residue Phe75 can be conservatively replaced by a tyrosine or histidine residue (residues are marked with an asterisk).
(TIFF)

Figure S2 **Sequence alignment of** ***Homo sapiens*** **and** ***Drosophila melanogaster*** **cytosolic 5′-nucleotidase IIIB with** ***Homo sapiens*** **cN-IIIA (isoform 2).** Identical residues are shown in white on red background, while similar ones are represented in red on white background. Secondary structure elements from the crystal structure of *^Dm^*cN-IIIB are shown above the alignment and residues are marked as in **Figure S1**. Note that Phe75 of *^Dm^*cN-IIIB is replaced by a tyrosine in *^Hs^*cN-IIIB and remarkably by a histidine in *^Hs^*cN-IIIA while the Trp121 of *^Dm^*cN-IIIB is replaced by a tyrosine in *^Hs^*cN-IIIA.
(TIFF)

Figure S3 **Overall structural comparison of substrate bound intracellular 5′-nucleotidases with known crystal structures.** The cytosolic 5′-nucleotidase II bound to IMP (PDB ID 2XCV), cN-IIIA bound to UMP (PDB ID 4FE3) and cN-IIIB bound to m^7^G (this study; PDB ID 4NV0) as well as the cytosolic 5′(3′)-deoxyribonucleotidase (cdN bound to dGMP; PDB ID 2JAO) and the mitochondrial-5′(3′)-deoxyribonucleotidase (mdN bound to dUMP; PDB ID 1Z4I) are shown. The HAD core domains are shown in blue colors (β-strands in light blue and α-helices in dark blue) and the cap domains in orange and yellow, respectively. Green spheres represent the magnesium ions and the bound substrates or products are shown as sticks (coloring as in **Figure 4**). Note that the HAD core domains are structurally similar in all of the structures while the cap domains are highly variable.
(TIFF)

Table S1 **Oligonucleotides used for site-directed mutagenesis.**
(PDF)

Movie S1 **Dynamic structural changes between open and closed conformations of cN-IIIA and cN-IIIB.** Trajectory connecting the crystal structures of the closed state of cN-IIIA bound to the substrate UMP (beginning of movie) and the open state of cN-IIIB bound to the reaction product 7-methyl guanosine (end of movie). The structure is shown in cartoon mode with substrates and products as well as the conserved Trp120 (cN-IIIB) or Trp113 (cN-IIIA) as sticks.
(MOV)
