# Supplementary figures and images for: Crystal Structures of the Novel Cytosolic 5′-Nucleotidase IIIB Explain Its Preference for m7GMP
Source: PLoS One. 2014 Mar 6;9(3):e90915. doi: 10.1371/journal.pone.0090915 (PMC3946280; doi:10.1371/journal.pone.0090915)

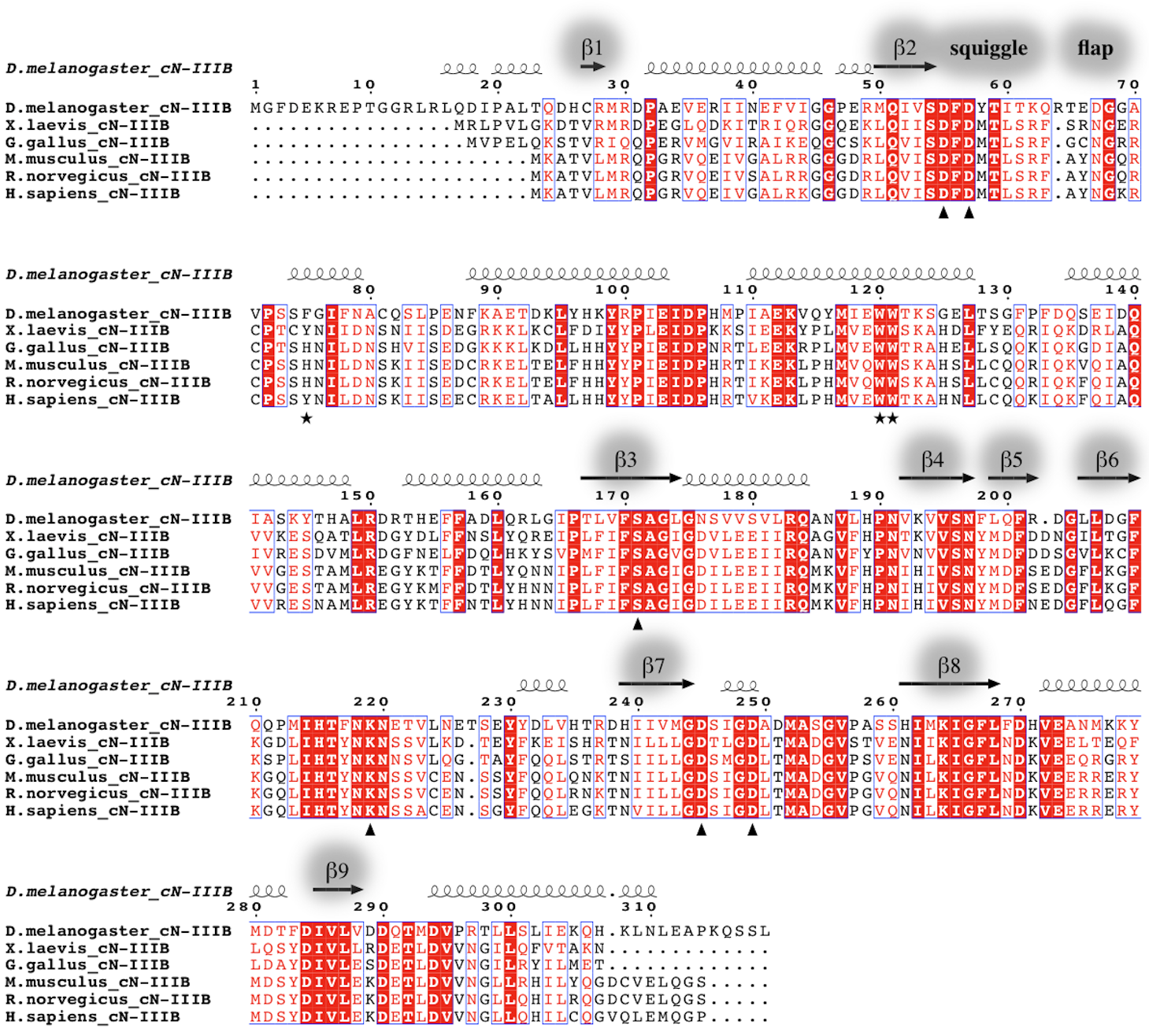

Supplement: Figure S1 — Multiple sequence alignment of cytosolic 5′-nucleotidase IIIB from Drosophila melanogaster , Xenopus laevis , Gallus gallus , Mus musculus , Rattus norvegicus and Homo sapiens . Identical residues are shown on red background, while similar ones are represented in red on white background. Secondary structure elements from the crystal structure of DmcN-IIIB are shown above the alignment. Note that important residues from motifs I-III (marked with a black triangle) as well as both tryptophans necessary for substrate binding in DmcN-IIIB are strictly conserved among all species while the second stacking residue Phe75 can be conservatively replaced by a tyrosine or histidine residue (residues are marked with an asterisk). (TIFF) [file pone.0090915.s001.tif]

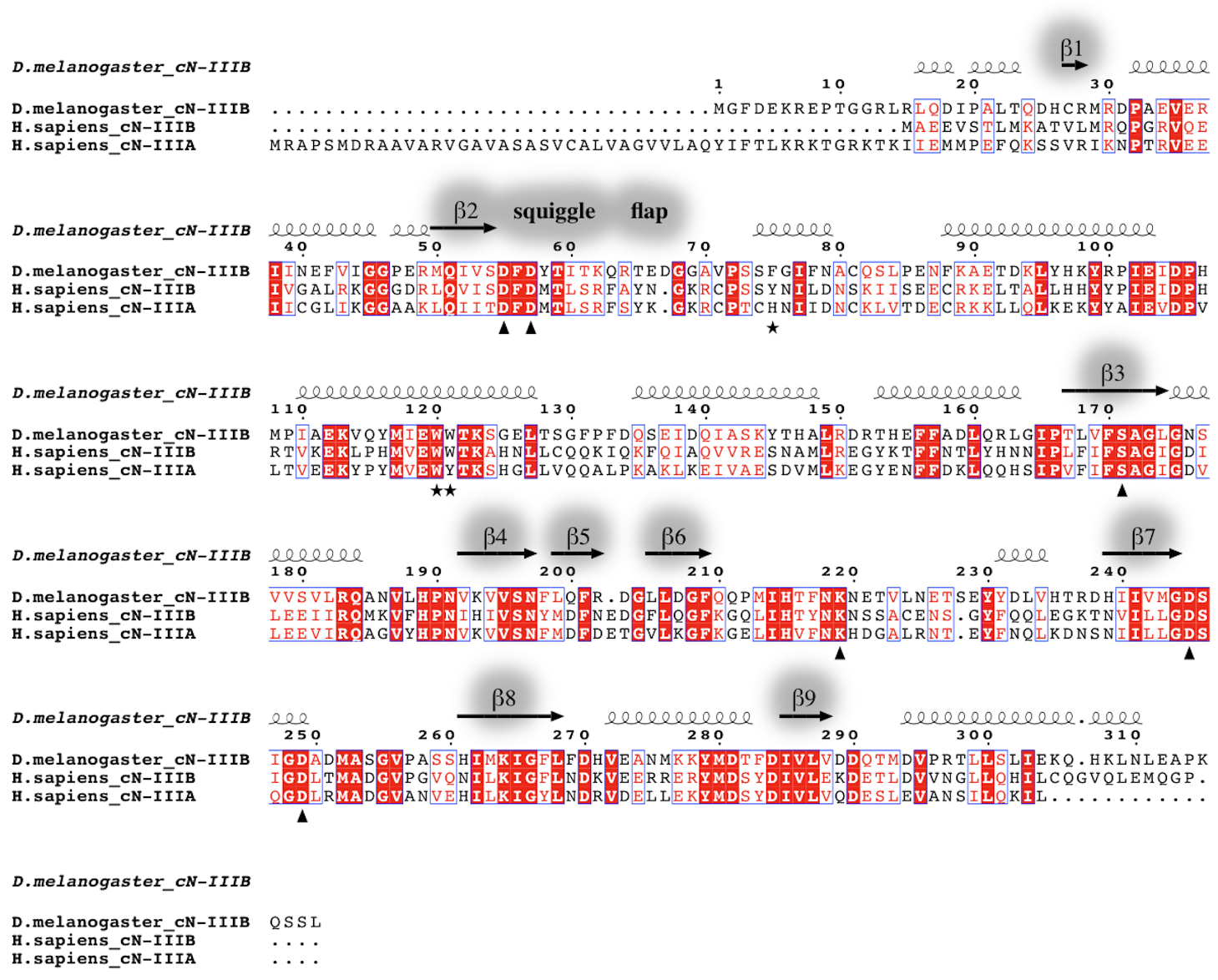

Supplement: Figure S2 — Sequence alignment of Homo sapiens and Drosophila melanogaster cytosolic 5′-nucleotidase IIIB with Homo sapiens cN-IIIA (isoform 2). Identical residues are shown in white on red background, while similar ones are represented in red on white background. Secondary structure elements from the crystal structure of DmcN-IIIB are shown above the alignment and residues are marked as in Figure S1. Note that Phe75 of DmcN-IIIB is replaced by a tyrosine in HscN-IIIB and remarkably by a histidine in HscN-IIIA while the Trp121 of DmcN-IIIB is replaced by a tyrosine in HscN-IIIA. (TIFF) [file pone.0090915.s002.tif]

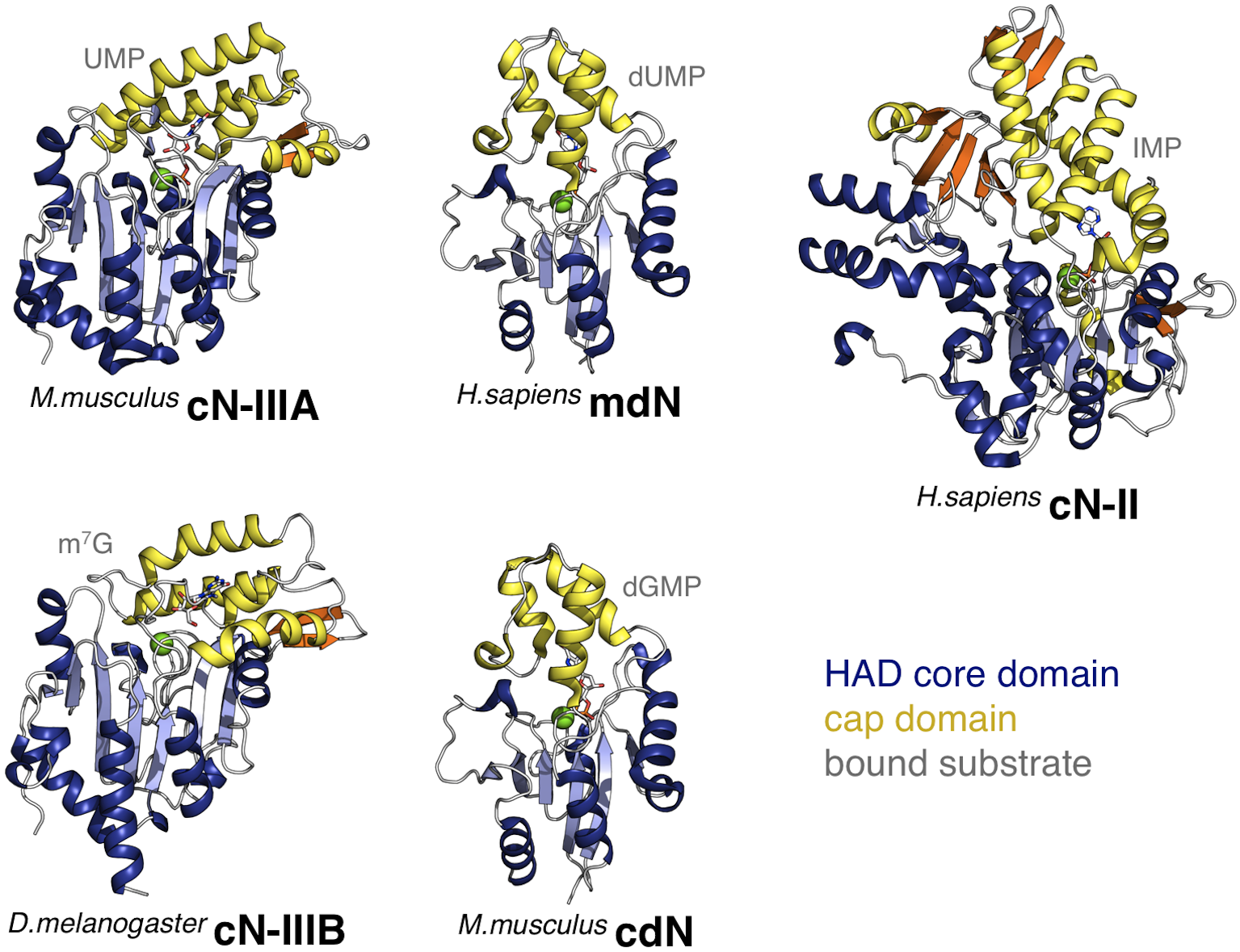

Supplement: Figure S3 — Overall structural comparison of substrate bound intracellular 5′-nucleotidases with known crystal structures. The cytosolic 5′-nucleotidase II bound to IMP (PDB ID 2XCV), cN-IIIA bound to UMP (PDB ID 4FE3) and cN-IIIB bound to m7G (this study; PDB ID 4NV0) as well as the cytosolic 5′(3′)-deoxyribonucleotidase (cdN bound to dGMP; PDB ID 2JAO) and the mitochondrial-5′(3′)-deoxyribonucleotidase (mdN bound to dUMP; PDB ID 1Z4I) are shown. The HAD core domains are shown in blue colors (β-strands in light blue and α-helices in dark blue) and the cap domains in orange and yellow, respectively. Green spheres represent the magnesium ions and the bound substrates or products are shown as sticks (coloring as in Figure 4 ). Note that the HAD core domains are structurally similar in all of the structures while the cap domains are highly variable. (TIFF) [file pone.0090915.s003.tif]
