# Supplementary material for: Crystal Structures of the Novel Cytosolic 5′-Nucleotidase IIIB Explain Its Preference for m7GMP
Source: PLoS One. 2014 Mar 6;9(3):e90915. doi: 10.1371/journal.pone.0090915 (PMC3946280; doi:10.1371/journal.pone.0090915)
Supplement: Table S1 — Oligonucleotides used for site-directed mutagenesis. (PDF) [file pone.0090915.s004.pdf]

Table S1: Oligonucleotides used for site-directed mutagenesis.

#	Construct	Orientation	Oligonucleotide sequence (5'→3')
1	F**N**WW	forward	agtgccctccagtttcaatatcttcaacgcctg
		reverse	caggcgttgaagatattgaaactggagggcact
2	**H**GWW	forward	gcagtgccctccagtcacgggatcttcaacgcctg
		reverse	caggcgttgaagatcccgtgactggagggcactgc
3	FGW**Y**	forward	gtacatgatcgagtggtataccaagtctggcgaactg
		reverse	cagttcgccagacttggtataccactcgatcatgtac
4	**H**GW**Y**		construct FGW**Y** with oligonucleotides of #2
5	F**N**W**Y**		construct FGW**Y** with oligonucleotides of #1
6	**HN**W**Y**		construct FGW**Y** with
		forward	ggagcagtgccctccagtcacaatatcttcaacgcctgcc
		reverse	ggcaggcgttgaagatattgtgactggagggcactgctcc
